# Lysosome-Associated Membrane Protein-3 (LAMP3) Expression in Oral Squamous Cell Carcinoma and Its Relationship With Clinicopathological Parameters: A Cross-Sectional Study

**DOI:** 10.7759/cureus.69790

**Published:** 2024-09-20

**Authors:** Marytresa S Jeyapriya, Sathish M Kumar, Madhavan R Nirmal

**Affiliations:** 1 Oral Pathology and Microbiology, Karpaga Vinayaga Institute of Dental Sciences, Chengalpet, IND; 2 Oral and Maxillofacial Pathology, Rajah Muthiah Dental College and Hospital, Chidambaram, IND

**Keywords:** cancer, cd208, dc-lamp, dendritic cells, lamp3, metastasis, oral cancer, oral squamous cell carcinoma, prognosis, tumor immune microenvironment

## Abstract

Background

Oral squamous cell carcinoma (OSCC) accounts for the majority of oral cancers globally. It is characterized by metastasis, poor prognosis, high recurrence rate, and poor five-year survival rate due to late detection or diagnosis at an advanced stage. Novel biomarkers that can predict the prognosis of patients with OSCC are needed to improve survival. Lysosome-associated membrane protein-3 (LAMP3) glycoprotein, a member of the LAMP protein family, is a molecular marker for mature dendritic cells. LAMP3 expression has been correlated with unfavorable prognosis in patients with various cancers. Few studies have examined the relationship between LAMP3 and clinicopathological parameters in OSCC. This study aims to analyze the immunohistochemical expression of LAMP3 in OSCC and its relationship with clinicopathological characteristics.

Methodology

In this study, 75 formalin-fixed, paraffin-embedded samples of cases diagnosed with primary OSCC were obtained and immunostained with LAMP3 antibody. Its expression was compared with clinicopathological parameters such as age, sex, tobacco and alcohol consumption habits, differentiation, TNM staging, tumor location, lymph node metastasis, lymphovascular invasion, perineural invasion, and pattern of invasion.

Results

Higher LAMP3 expression was highly significantly associated with the TNM stage (p = 0.001). High expression of LAMP3 was significantly associated with T stage (p = 0.002) and lymph node metastasis (p = 0.002). All poorly differentiated OSCC cases (n = 2, 100%) showed a high expression of LAMP3.

Conclusions

High LAMP3 expression and its significant association with TNM stage, T stage, and lymph node metastasis suggest a potential role for LAMP3 in OSCC carcinogenesis. High LAMP3 expression in poorly differentiated OSCC might indicate that it plays a pivotal role in oncogenic cell transformation. Our results indicate that LAMP3 may be a predictive marker for poor prognosis in OSCC.

## Introduction

Oral squamous cell carcinoma (OSCC) constitutes a major subgroup of head and neck carcinomas accounting for 90% of all malignancies worldwide. Over 300,000 new cases of OSCC are diagnosed across the globe annually and the incidence rate is on the rise [1,2]. OSCC occurs at a high frequency in India [3]. OSCC is often associated with metastasis, poor prognosis, high recurrence rate, and poor five-year survival rate owing to late detection or diagnosis at an advanced stage. Unique molecular markers that can predict the prognosis of patients with OSCC are needed to improve clinical outcomes [2].

Lysosome-associated membrane protein-3 ( LAMP3) glycoprotein is a member of the LAMP protein family. It is a molecular marker of mature dendritic cells (CD208, DC-LAMP). LAMP proteins are glycosylated type 1 integral membrane proteins that reside in lysosomal membranes [2,4]. LAMP3 induces and promotes the migration and invasion of tumor cells [1,2,5]. High LAMP3 expression is correlated with unfavorable prognosis in patients with various cancers, including head and neck squamous cell carcinomas [6]. Numerous studies have reported a significant relationship between LAMP3 and lymph node metastasis [5]. A strong association has been reported between LAMP3 expression and the promotion of metastatic potential in in-vivo and in-vitro studies [7]. LAMP3 may be a potential biomarker for the survival of cancer patients [6]. Although LAMP3 expression and its role have been studied in various cancers, limited studies have examined the relationship between LAMP3 and clinicopathological parameters in OSCC. Hence, this study aimed to analyze the immunohistochemical expression of LAMP3 in OSCC and its relationship with clinicopathological characteristics.

## Materials and methods

Tissue samples and patient characteristics

This study was approved by the institutional human ethics committee (approval number: KIDS/IEC /009/2021/IV) This study was performed using archived formalin-fixed, paraffin-embedded tissue samples obtained from 75 patients with OSCC who underwent surgery between 2018 and 2021 at Karpaga Vinayaga Institute of Dental Sciences, Chengalpet. Clinicopathological data, including age, sex, tobacco and alcohol consumption habits, differentiation, TNM staging, tumor location, lymph node metastasis, lymphovascular invasion, perineural invasion, and pattern of invasion, were obtained from clinical records. The inclusion criterion was primary oral cancer in the oral cavity. The exclusion criterion was prior radiotherapy or chemotherapy in the head and neck area. The World Health Organization system was used to perform histological grading of the tumors. Clinical staging was performed according to the eighth edition of the American Joint Committee on Cancer classification. The sample size was calculated using G*Power software (version 3.1.9.4). Using a mean difference between two independent groups, an error probability of 0.05, a study power of 0.8, and an effect size of 0.33, a minimum sample size of 73 was calculated, which was rounded to 75.

Tissue preparation and Immunohistochemistry

In this study, 4 µm thick sections of formalin-fixed, paraffin-embedded tumor specimens from OSCC patients were used for immunohistochemical analysis. Sections were taken onto positively charged slides (Pathnsitu Biotechnologies, USA). The sections were then deparaffinized and rehydrated. They were then subjected to heat-induced antigen retrieval using citrate buffer at a pH of 6.0. The tissues were then blocked for endogenous peroxidase activity with pre-diluted 3% hydrogen peroxide, and incubated with 1:200 dilution of anti-LAMP3 mouse monoclonal primary antibody (anti-LAMP3, clone 16H11.2, Merck Millipore, Germany). Later, the sections were incubated with a poly excel HRP/DAB secondary antibody system (pre-diluted, Pathnsitu Biotechnologies, USA) for 30 minutes, followed by counterstaining with Mayer’s hematoxylin. Human normal oral mucosa were used as positive controls.

Immunohistochemical evaluation

**Figure 1:**
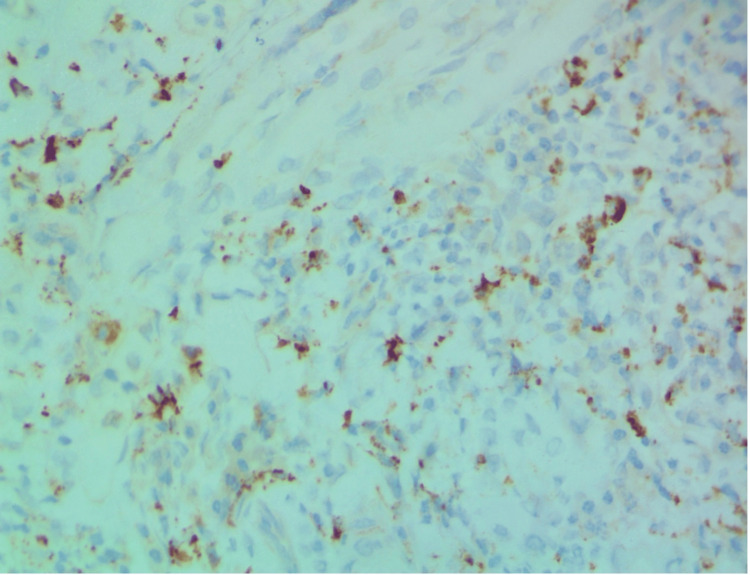
High-power (40×) view showing LAMP3 expression.

Two trained and independent observers, blinded to the clinical outcomes, interpreted the slides. The expression of LAMP3 cells was evaluated using the scoring system reported by Lu et al. [2]. The scoring for a percentage of LAMP3-positive cells was done as follows: a score of 0 for 0% staining, 1 for 1-33% staining, 2 for 34-66%, and a score of 3 for 67-100% staining. The intensity of LAMP3 staining was scored as follows: 0 indicated no staining, 1 weak staining, 2 moderate staining, and 3 strong staining. The final staining score (ranging from 0% to 234%) was the product of the percentage and intensity scores. The mean value was 73%. Samples with scores higher than 73% were classified as having high expression and those lesser than 73% were classified as having low expression (Figure 1).

Statistical analysis

Data were compiled in Microsoft Excel (Microsoft Corp., Redmond, WA, USA), and statistical analyses were performed using SPSS version 22 (IBM Corp., Armonk, NY, USA). Pearson’s chi-square test was used to evaluate the association between LAMP3 expression and clinicopathological parameters. P-values <0.05 were considered to be statistically significant.

## Results

Study participants, demographic details, and histopathological features

Of the 75 patients included in this study, 49 were males and 26 were females, with ages ranging from 31 to 80 years. Overall, 56 cases were well differentiated, 17 cases were moderately differentiated, and two were poorly differentiated. Stage I cancer was seen in 13 patients, stage II cancer in 20 patients, stage III cancer in 27 patients, and stage IVA cancer was seen in 15 patients. Twelve patients had lymph node metastases. Further demographic details and clinicopathological features are summarized in Table 1.

**Table 1:** Relationship of LAMP3 expression with clinicopathological parameters in oral squamous cell carcinoma. *: p-value <0.05* = Statistically significant; **: p-value <0.001 = highly significant.

Variables		n	Low expression		High expression		Pearson’s chi-square	P-value
			n	%	n	%		
Total		75	31	41.40%	44	58.60%		
Gender	Male	49	20	40.80%	29	59.20%	0.016	0.901
	Female	26	11	42.30%	15	57.70%		
Age (years)	31–50	29	11	37.90%	18	62.10%	4.746	0.093
	51–70	40	15	37.50%	25	62.50%		
	>71	6	5	83.30%	1	16.70%		
Tobacco smoking/chewing	Absent	38	17	44.70%	21	55.30%	0.379	0.827
	Tobacco smoking present	32	12	37.50%	20	62.50%		
	Tobacco chewing present	5	2	40.00%	3	60.00%		
Alcohol	Absent	32	13	40.60%	19	59.40%	1.262	0.532
	Present less than 1 times weekly	15	8	53.30%	7	46.70%		
	Present more than 1 times weekly	28	10	35.70%	18	64.30%		
Tumor site	Buccal mucosa	24	7	29.10%	17	70.90%	13.442	0.144
	Tongue	22	10	45.40%	12	54.60%		
	Mandible	1	0	0.00%	1	100.00%		
	Lip	9	6	66.60%	3	33.30%		
	Palate	2	2	100.00%	0	0.00%		
	Retromolar trigone	7	4	57.10%	3	42.90%		
	Gingiva	1	1	100.00%	0	0.00%		
	Floor of the mouth	5	1	20.00%	4	80.00%		
	Angle of the mouth	2	0	0.00%	2	100.00%		
	Hypopharynx	2	0	0.00%	2	100.00%		
Histopathological grade	Well-differentiated squamous cell carcinoma	56	23	41.00%	33	59.00%	1.64	0.44
	Moderately differentiated squamous cell carcinoma	17	8	47.00%	9	53.00%		
	Poorly differentiated squamous cell carcinoma	2	0	0.00%	2	100.00%		
TNM stage	Stage I	13	10	76.90%	3	23.10%	47.063	0.001**
	Stage II	20	18	90.00%	2	10.00%		
	Stage III	27	3	11.10%	24	88.90%		
	Stage IVA	15	0	0.00%	15	100.00%		
T stage	T1	15	11	73.30%	4	26.70%	17.405	0.002*
	T2	37	18	48.60%	19	51.40%		
	T3	19	2	10.50%	17	89.50%		
	T4	2	0	0.00%	2	100.00%		
	T5	2	0	0.00%	2	100.00%		
Lymph node metastasis	Absent	63	31	49.20%	32	50.80%	10.065	0.002*
	Present	12	0	0.00%	12	100.00%		
Lymphovascular invasion	Absent	67	30	44.70%	37	55.30%	3.07	0.08
	Present	8	1	12.50%	7	87.50%		
Perineural invasion	Absent	65	28	43.00%	37	57.00%	0.611	0.434
	Present	10	3	30.00%	7	70.00%		
Pattern of invasion	Cohesive	32	13	40.60%	19	59.40%	1.822	0.402
	Non-cohesive	34	16	47.00%	18	53.00%		
	Dispersive	9	2	22.20%	7	78.80%		

Distribution of LAMP3-positive dendritic cells in OSCC tissues

Immunohistochemical staining showed that LAMP3 primarily presented as brown cytoplasmic staining dendritic cells. Positively stained cells were predominantly located in the invasive tumor front, as described in previous literature [8] (Figure 1).

Relationship between LAMP3 expression and clinicopathological parameters

Higher LAMP3 expression was highly significantly associated with the TNM stage (p = 0.001). High expression of LAMP3 was significantly associated with T stage (p = 0.002) and lymph node metastasis (p = 0.002) (Table 1). All poorly differentiated OSCC cases (n = 2, 100%) showed a high expression of LAMP3. However, there was no statistical significance between LAMP3 expression and age, gender, tobacco usage, alcohol consumption, tumor site, lymphovascular invasion, perineural invasion, and pattern of invasion.

## Discussion

Lysosomes are small membrane-bound vesicles responsible for the biodegradation of macromolecules in cells. They regulate cellular homeostasis and their dysregulation can lead to chronic diseases. In cancer cells, lysosomal biogenesis and autophagy are augmented to cope with increased metabolism and growth in hypoxic tumor microenvironments. They participate in cell signaling and facilitate tumor progression [9].

Lysosomes are usually dispersed throughout the cytoplasm in normal cells. In cancer cells, lysosomes migrate toward the cell periphery owing to the acidic pH of the tumor microenvironment. This centrifugal transport leads to the secretion of acid hydrolases into the extracellular space, which, in turn, degrades the extracellular matrix. This results in the migration and invasion of cancer cells. During carcinogenesis, lysosomes also undergo changes in their number, morphology, pH, and hydrolase content [10].

LAMPs are a series of highly glycosylated proteins present in lysosomal membranes. These include LAMP1, LAMP2, LAMP3, CD68/macrosialin/LAMP4, and BAD-LAMP/LAMP5 proteins. LAMP proteins can relocate to the cell membrane surface in cancer cells. LAMP3, the third member of this family of proteins, is a molecular marker for mature dendritic cells. It is known by other names such as DC-LAMP and CD208. It is a newly discovered, tumor-specific, and hypoxia-induced protein. The gene encoding this protein is located on the chromosome 3q segment. High LAMP3 expression is associated with lymph node metastasis and poor overall survival in patients with gastrointestinal cancer, breast cancer, and esophageal cancer [1,6,11].

In this study, we investigated the expression of LAMP3 in OSCC and its association with clinicopathological parameters. The study revealed that higher LAMP3 expression was highly significantly associated with the TNM stage. This is similar to a study by Sun et al., where high LAMP3 expression was significantly associated with tumor stage in gastrointestinal cancer and colorectal cancer [4]. Similar results were reported in a study by Lu et al., where high expression of LAMP3 was significantly linked to tumor stage in OSCC [2]. The advanced TNM stage was associated with poor prognosis. Increased expression of LAMP3 is reported to be associated with unfavorable prognosis of patients with various cancers such as laryngeal squamous cell carcinoma, gastrointestinal tumors, breast cancers, and cervical cancer [5-7,11,12]. This might be because cancer aggressiveness is increased by the lysosomal release of LAMP3 [13].

In this study, high LAMP3 expression was significantly linked to T stage and lymph node metastasis. This is similar to a study by Sun et al., which reported that high LAMP3 expression was significantly associated with lymph node metastasis in gastrointestinal cancer [4]. Kanao et al., in their study of uterine cervical cancer, concluded that overexpression of LAMP3 was significantly associated with metastatic potential [5]. In a similar study, Wang et al. demonstrated that high cytoplasmic expression of LAMP3 was significantly linked to metastasis [14]. Similarly, Nagalkerke et al. reported high LAMP3 expression and promotion of metastasis in breast carcinoma [7]. LAMPs have been linked to the metastatic potential of tumors via many mechanisms. They bear sialylated Lewis X antigen. Cancer cells overexpress this antigen and attach with E-selectins and endothelial cells which leads to metastasis [7]. LAMP3 also regulates metastasis through a hypoxia-mediated mechanism. Hypoxia has been associated with aggressive and metastatic cancers, and several hypoxia-regulated genes initiate dissociation and migration of cells, thereby regulating tumor metastasis [15]. A hypoxic tumor microenvironment may lead to high expression of LAMP3 via an unfolded protein response. This is an adaptive response to endoplasmic reticulum stress. Thus, LAMP3 might play a key role in metastasis [7,12,16]. LAMP3 is considered a downstream target gene of the tumor suppressor gene *TP53* [11]. These findings indicate that LAMP3 might play a potential role in the metastasis of OSCC.

Poorly differentiated carcinomas are associated with a poor prognosis [17]. LAMP3 correlated with poor prognosis in many cancers [5-7,11,12]. In this study, all poorly differentiated carcinomas showed high expression of LAMP3. Similar results were reported by Lu et al., who demonstrated that high LAMP3 expression was significantly associated with the degree of differentiation in OSCC [2]. This could be because during carcinogenesis, lysosomes undergo changes in their morphology, luminal pH, number, hydrolase content, and intracellular distribution as a result of acidification of the tumor microenvironment, eventually leading to oncogenic transformation of the cells [18,19]. LAMP3, a lysosomal protein, might play a significant role in oncogenic transformation.

This study has a few limitations. First, in this study, a comparatively smaller sample size was investigated. However, in future studies, the use of a larger sample size along with analysis of the overall survival rate and five-year survival rate may yield a more elaborate result and elucidate whether LAMP3 may be a prognostic marker and anti-cancer target in OSCC.

## Conclusions

This study investigated the immunohistochemical expression of LAMP3 in OSCC and its relationship with clinicopathological characteristics. High LAMP3 expression and its significant association with TNM stage, T stage, and lymph node metastasis suggest a major role for LAMP3 in OSCC progression. High LAMP3 expression in poorly differentiated OSCC might indicate that LAMP3 plays a pivotal role in oncogenic cell transformation. Our results indicate that LAMP3 may be a predictive marker for poor prognosis in OSCC.
